# Effect of Physical Structures of Food Matrices on Heat Resistance of *Enterococcus faecium* NRRL-2356 in Wheat Kernels, Flour and Dough

**DOI:** 10.3390/foods9121890

**Published:** 2020-12-18

**Authors:** Biying Lin, Yufei Zhu, Lihui Zhang, Ruzhen Xu, Xiangyu Guan, Xiaoxi Kou, Shaojin Wang

**Affiliations:** 1 College of Mechanical and Electronic Engineering, Northwest A&F University, Yangling 712100, China; linbiying@nwafu.edu.cn (B.L.); 1000005296@ujs.edu.cn (L.Z.); ruzhen_xu@nwafu.edu.cn (R.X.); xiangyuguan@nwafu.edu.cn (X.G.); kouxiaoxi@nwsuaf.edu.cn (X.K.); 2 College of Animal Science and Technology, Northwest A&F University, Yangling 712100, China; zhuyufei@nwsuaf.edu.cn; 3 Department of Biological Systems Engineering, Washington State University, Pullman, WA 99164-6120, USA

**Keywords:** wheat, physical structure, Weibull model, heat resistance, microstructure, surrogate microorganisms

## Abstract

Nonpathogenic surrogate microorganisms, with a similar or slightly higher thermal resistance of the target pathogens, are usually recommended for validating practical pasteurization processes. The aim of this study was to explore a surrogate microorganism in wheat products by comparing the thermal resistance of three common bacteria in wheat kernels and flour. The most heat-resistant *Enterococcus faecium* NRRL-2356 rather than *Salmonella* cocktail and *Escherichia coli* ATCC 25922 was determined when heating at different temperature–time combinations at a fixed heating rate of 5 °C/min in a heating block system. The most heat-resistant pathogen was selected to investigate the influences of physical structures of food matrices. The results indicated that the heat resistance of *E. faecium* was influenced by physical structures of food matrices and reduced at wheat kernel structural conditions. The inactivation of *E. faecium* was better fitted in the Weibull distribution model for wheat dough structural conditions while in first-order kinetics for wheat kernel and flour structural conditions due to the changes of physical structures during heating. A better pasteurization effect could be achieved in wheat kernel structure in this study, which may provide technical support for thermal inactivation of pathogens in wheat-based food processing.

## 1. Introduction

Many studies have reported that wheat and its related products have been contaminated with *Salmonella* [1,2,3,4]. These contaminations threaten public health, although the bacteria are mainly located at surfaces of wheat kernels at very low levels with 0.110 ± 0.448 MPN/g *Salmonella* cells [4]. A series of risks of an increasing microbial level still exist due to cross-contamination between source materials and the external environment during harvesting, storage, and processing [4,5,6]. In addition, a larger sanitary challenge is faced in wheat-based food due to mixing with meat, egg, nuts, or other microbial carriers [7,8], and consumption of not fully cooked foods [9,10]. In the process of wheat source material to wheat-based food, there are three physical structures, including wheat kernels (graininess), wheat flour (powder) and wheat dough (paste). These structural changes would affect the efficiency of the microbial inactivation during different processing practices. An efficient pasteurization method corresponding to changes in physical structures of wheat source materials is urgently needed.

Heat treatment is one of the traditional microbial control methods in agricultural and food processing applications. There are many factors influencing microbial inactivation, such as temperature, water activity (a_w_), heating rate and physical structure of food matrices [11,12,13,14,15,16]. Decimal reduction time (*D*-value), the time (min) required to reduce the microbial population by 10-fold (1 log) at a constant temperature (°C), is used to measure microbial heat resistance. A larger *D*-value indicates that the microorganisms are harder to inactivate at a fixed temperature. Except for the obtained knowledge so far that heat resistance of microorganisms could be reduced with increasing of temperature and a_w_ [17,18,19], component and physical structure of the food matrices may also influence the microbial inactivation levels. For instance, D_80 °C_-values of 6.9 and 17.0 min for *Salmonella enteritidis* PT 30 are observed in wheat flour and peanut butter, respectively, at a_w_ of 0.45 at 20 °C due to changes of a_w_ at high temperatures (80 °C) derived from various main components in food matrices, such as starch in flour and fat in peanut butter [13]. Although some materials have the same compositions, D_80 °C_-values of 33.4 and 18.3 min for *S*. *enteritidis* PT 30 are found in almond meal and butter, respectively, at a_w_ of 0.41, mainly owing to the different physical structures between powder and paste [12]. In addition, the difference in particle sizes also affects the heat resistance of microorganisms [12,20]. These variations in microbial heat resistance may be attributed to differences in the microbial survival environments in food matrices with their different physical structures [11,21]. During the process from wheat-to-wheat-based food, physical structure variations with graininess, powder and paste may affect the heat resistance of microorganisms. Studies on the effective bactericidal data in wheat-based products with various physical structures may provide technical guidance for developing effective industrial pasteurization processes.

Thermal inactivation kinetic study of microorganisms can provide solid and reliable thermal processing parameters (heating temperature and time) for the food industry. The linear model described by first-order kinetics is widely used to determine the *D*-value for microorganisms, which can accurately predict the linear microbial inactivation curve [16,22,23]. In addition, the Weibull model has been successfully applied to better fit nonlinear microbial inactivation curves [24,25,26,27,28,29]. A better temperature and heating uniformity control for each kinetic inactivation can make the fitting equation more accurate. Thus, a thermal death time (TDT) heating block system (HBS) was selected to study the thermal inactivation kinetics of microorganism in this study since it has been successfully applied to determine thermal responses of microorganisms in liquid, semi-solid and solid foods [30,31]. The TDT HBS consists of a heating unit with six cells, a data acquisition/control unit, and a computer. The heating rate, heating temperature and holding time of the materials in cells of the heating unit are controlled by Visual Basic software and two proportional-integral-derivative (PID) controllers (I32, Omega Engineering, Inc., Stamford, CT, USA) according to the preset heating conditions.

A nonpathogenic surrogate microorganism is suggested to be applied in practical microbial validations [18,32,33,34]. Many studies show that *Enterococcus faecium* NRRL-2356 and *Escherichia coli* ATCC 25922 are used as a surrogate for *Salmonella* in wheat flour [33], almond [32,35,36], pepper [20,34] and peanut [35]. A nonpathogenic microorganism with a similar or higher heat resistance should be used as a surrogate microorganism. However, no comparison data of heat resistances between *E. faecium* NRRL-2356 and *E. coli* ATCC 25922 have been reported in wheat with different physical structures.

The objectives of this study were (1) to determine the most heat-resistant strain when inoculated in wheat kernels and flour under three temperature–time combinations using the TDT HBS, (2) to study the influences of physical structures of food matrices on the heat resistance of the most heat-resistant strain, and (3) to evaluate the influence of the physical structure of food matrices on the morphological change and heat resistance of the most heat-resistant strain under the given heating conditions by modified Weibull model and scanning electron microscope observation.

## 2. Materials and Methods

### 2.1. Materials

Wheat kernels (*Triticum aestivum* L.) (length: 6.60 ± 0.32 mm, width: 3.46 ± 0.26 mm, and thickness: 2.91 ± 0.19 mm) were obtained from a local farmer in Yangling, Shaanxi Province, China. The wheat samples were stored in sealed polyethylene bags at 4 ± 1 °C prior to the tests.

*S*. *typhimurium* ATCC 50115, *S*. *braederup* H9812 and *E. coli* ATCC 25922 were acquired from the College of Food Science and Engineering, Northwest A&F University (Yangling, China). Three bacteria strains were kept at −20 °C in Luria–Bertani broth supplemented with 20% (*v/v*) glycerol. *E. faecium* NRRL-2356 was acquired from the College of Food Science and Technology, Sichuan Agricultural University (Ya’an, China) and kept at −20 °C in tryptic soy broth supplemented with 0.6% (*w/v*) yeast extract and 20% (*v*/*v*) glycerol.

Luria–Bertani broth, Luria–Bertani agar, tryptic soy broth and tryptic soy agar were purchased from Beijing Land Technology Co., Ltd. (Beijing, China). Yeast extract, peptone and saline were purchased from Beijing Aoboxing Biotechnology Co., Ltd. (Beijing, China).

### 2.2. Sample Preparation and Characteristics

To study the influences of different physical structures of food matrices on the heat resistance of bacteria, wheat samples at graininess, powder and paste structures were prepared. For comparing the microbial heat resistance, a key is to control the a_w_ level of food matrices. However, graininess, powder and paste structures cannot exist at the same a_w_ of food matrices. The influence of different physical structures of food matrices on the heat resistance of bacteria was conducted at two a_w_ levels. According to Syamaladevi et al. [13], wheat flour had a wide a_w_ range between 0.10 to 0.85 corresponding to moisture content (MC) from 7.50 to 19.00% wet basis (w.b.) at 20 °C; thus, wheat kernels at original MC of 11.25% w.b. (a_w_ = 0.66) and its milled flour (particle size: −16 mesh) were selected to study the influence of graininess and powder structures of food matrices on the heat resistance of *E. faecium*. The prepared wheat kernels and flour were conditioned in closed containers at 4 °C for at least 2 d for equilibrium and then were sterilized by autoclaving at 105 °C for 10 min to achieve a background bacteria level below detection (<1 log CFU/g) without any damage to the materials [23,37,38].

Wheat dough (38.50% w.b., a_w_ = 0.99) was prepared by combining it with sterile water at minimum water addition content for dough formation and gently mixing by hand for 5 min, equilibrating for 15 min in the biosafety hood. The minimum water addition content was determined by preliminary tests. Wheat kernel samples were prepared by added pre-calculated weight of sterile distilled water into presterilized wheat kernels and sealed in sterile bags, equilibrating at 4 °C for 2 d, and were gently hand mixed every 2 h until the final a_w_ was equal to that of dough. The wheat kernels with adjusted moisture levels and dough were selected to study the influence of graininess and paste structures of food matrices on the heat resistance of *E. faecium*. The background bacteria concentrations of the prepared materials with readjusted moisture levels were determined to be less than 1 log CFU/g by plate count method before the HBS heating experiment.

The MC and a_w_ of samples were determined at 105 °C using the oven method and measured at 25 °C by an Aqua Lab water activity meter (Aqua Lab 4 TE, Decagon Devices, Inc., Pullman, WA, USA), respectively. Densities of wheat kernels, wheat flour and dough in the cell (inner diameter: 20.6 mm, height: 3 mm) of HBS were measured by the liquid displacement method [39] and according to the reference [40], respectively. MC, a_w,_ and densities of wheat kernels, wheat flour and dough are shown in Table 1, and MC and a_w_ showed no significant difference between two physical structures of food matrices (*p* > 0.05). A maximum a_w_ difference less than 0.015 in materials indicated that the influence of a_w_ could be ignored when comparing the heat resistance of bacteria in food matrices within two physical structures [41].

### 2.3. Preparation and Inoculation of Cell Suspension

Each culture was prepared by subjecting to two consecutive transfers (24 h incubation periods at 37 °C) in 30.0 mL and 300.0 mL Luria–Bertani broth for *Salmonella* and *E. coli*, and tryptic soy broth supplemented with 0.6% (*w/v*) yeast extract for *E. faecium*, respectively [15,20,33]. Two *Salmonella* cultures were mixed to form a 2-strain cocktail of *Salmonella*. The bacterial suspensions were centrifuged at 3500 r/min, and their final concentrations were adjusted to 10^11^–10^12^ CFU/mL with 200 μL 8.5% saline for *Salmonella* cocktail and *E. coli* [20], and 0.1% peptone water for *E. faecium* [15] used in subsequent experiments and refrigerated for no more 2 d prior to inoculation in materials.

For comparing the influence of graininess and powder structures of food matrices on the heat resistance of *E. faecium*, an aliquot of 10 μL bacteria suspension was inoculated in ~0.7 g wheat kernels and flour by gently hand mixing for 1 min and then dried for ~40 min inside a biosafety hood until the a_w_ of the sample equaled to that of a non-inoculated sample [12,15]. By simulating bacterial liquid distribution with the blue ink, the inoculation uniformity was determined by the color distribution with photos that the deviations of the L* and b* values were less than 8% and 7%, respectively, according to preliminary tests.

To investigate the effect of graininess and paste structures of materials on most heat-resistant bacteria, about 0.9 g wheat kernels and dough were inoculated by the same steps as described above. The drying time was determined by preliminary tests according to the a_w_ of the sample using the water activity meter [2,12,15]. After equilibrating, the inoculated samples were transferred into TDT cells and closed immediately.

### 2.4. Heat Treatment

Heat treatments of *E. faecium*, *Salmonella* cocktail, and *E. coli* when inoculated in materials with three physical structures were conducted in the TDT HBS, which can precisely control the heating rate, set-point temperature and holding time in inactivation kinetic experiments [15,31]. In HBS, six TDT cells (inner diameters: 20.6 mm and height: 3 mm) were designed to hold a small weight capacity to achieve heating uniformity in materials [31]. One TDT cell was filled with uninoculated material to monitor temperature changing, while the other five TDT cells were filled with inoculated samples to perform the heat inactivation of bacterial. The detailed information about TDT HBS can be found in other studies [30,31]. In this study, all heating conditions were conducted at a fixed heating rate of 5 °C/min to simulate the fast-heating rate commonly used in radio frequency pasteurization treatments [33,42].

#### 2.4.1. Comparison of Microbial Heat Resistances among *E. faecium*, *Salmonella* Cocktail, and *E. coli*

Various microbial strains may respond to heat differently. The safe treatment protocol in the laboratory should be developed based on the most heat-resistant strain. Since the structure change from flour to dough is caused by MC changing, wheat kernel (a_w_ = 0.66) and flour (a_w_ = 0.65) were selected as the representative model to compare the relative heat-resistance among *E. faecium*, *Salmonella* cocktail, and *E. coli* in food matrices using the HBS regardless of physical structures of food matrices in this experiment. Six temperature and time combinations of 75 °C + 2 min, 80 °C + 0.8 min and 85 °C + 0.2 min in wheat kernels, or 75 °C + 4 min, 80 °C + 1.5 min and 85 °C + 0.5 min in wheat flour were selected to determine the most heat-resistant strain according to preliminary tests.

#### 2.4.2. Thermal Inactivation Kinetics

The most heat-resistant strain was further used to study the influences of different physical structures of food matrices on the heat resistance of bacteria in the HBS. Temperatures of 75, 80 and 85 °C or 60, 62, and 64 °C were chosen to compare the influence of graininess and powder or paste structures of food matrices on the heat resistance of bacteria. Different temperature and time combinations were selected to achieve about 4-log CFU/g reductions during holding time according to preliminary tests.

### 2.5. Enumeration

The TDT cell pulled out when the sample temperature reached the target set-point was defined as a time zero sample, and the remaining cells were removed at five different holding time intervals. All the cells were cooled in 4 °C ice water immediately. To enumerate strain survivors, treated samples after cooling were transferred into 50 mL centrifuge tubes, diluted 1:10 with 8.5% saline for *Salmonella* cocktail and *E. coli* [20] and 0.1% peptone water for *E. faecium* [33], respectively. Appropriate tenfold serial dilutions were spread-plated in duplicate onto Luria–Bertani agar for *Salmonella* and *E. coli* [20] and tryptic soy agar supplemented with 0.6% (*w/v*) yeast extract for *E. faecium* [33], respectively. The plates were incubated aerobically at 37 °C for 48 h, then the colonies were counted, and the populations were converted to log CFU/g.

### 2.6. Fitting of Kinetic Inactivation

The primary models, first-order kinetic and Weibull distribution models, were used to describe inactivation curves. The first-order kinetic model is described below [15,23]:

(1) $$\log\frac{N_{t}}{N_{0}}=-\frac{t}{D}$$

where *N_t_* and *N*_0_ are microbial populations (CFU/g) at isothermal treatment time *t* and time 0 (min) when subjected to a target temperature; *D* is the time (min) required to reduce the microbial population by 10-fold (1 log) at a constant temperature (°C) [43].

The Weibull distribution model is described as follows [20,29]:

(2) $$\log\frac{N_{t}}{N_{0}}=-{\left(\frac{t}{\delta }\right)}^{p}$$

where *δ* is the time (min) for the first decimal reduction at a given temperature, and *p* is the shape parameter, dimensionless, which describes whether the fitting curve is linear (*p* = 1) or nonlinear (*p* ≠ 1) with a decreasing (*p* < 1) or increasing (*p* > 1) inactivation rate with time.

As recommended by references [20,26,44], to eliminate influences of the environmental factors on the shape parameter of the Weibull model, a method with standardization of *p*-value in a data set was used in this study. It means that a single *p*-value was estimated from the whole set of survival curves of bacterial, and the *δ*’-values were re-estimated at a fixed *p*-value.

To compare the goodness of fit between linear and Weibull models, the Akaike information criterion (*AIC*) was successfully applied in evaluating various microbial fitting models and can be calculated as follows [45,46]:

(3) $$AIC=n\ln\left(\frac{RSS}{n}\right)+2(p+1)+\frac{2(p+1)(p+2)}{n-p-2}$$

where *RSS* is residual sums of squares, *n* stands for the number of measurements, and *p* for the number of parameters. The model with the lowest *AIC* number performs the best.

### 2.7. Microstructure Analysis

To compare the influence of the physical structure of food matrices on wheat samples and the most heat-resistant strain inoculated in wheat samples, wheat kernels (a_w_ = 0.66) and flour were treated in the HBS at 80 °C for 5 min. Wheat kernels (a_w_ = 0.99) and dough were treated in the HBS at 62 °C for 5 min. After heat treatment, all five TDT samples were cooled in ice water (<4 °C) for at least 2 min.

Scanning electron microscope (SEM) (Nano SEM 450, FEI, Hillsboro, OR USA) was used to investigate the microstructure of wheat samples and *E. faecium* inoculated in wheat samples affected by different physical structures of food matrices before and after heat treatment. Control samples without any treatment and treated samples randomly collected from five mixing TDT samples were prepared for SEM observations. For microstructure analysis of wheat samples, wheat kernel surfaces were cut into thin slices (5 × 5 × 3 mm^3^) and dried in a drying oven at 40 °C for 24 h. Wheat doughs were cut into thin slices (5 × 5 × 3 mm^3^) and dried by freeze dryer (FreeZone Plus 2.5 L, LABCONCO, Kansas, MO, USA) overnight. For microstructure analysis of *E. faecium*, cells inoculated in wheat kernel surfaces, flour and dough were fixed in 2.5% glutaraldehyde at 4 °C overnight. Then, samples were washed twice with phosphate buffer (0.1 M, pH 7.2) for 10 min each time. Samples were dehydrated in a graded series of ethanol (30%, 50%, 70%, 80%, 90%, 100%, and 100% (*v/v*)) for 10 min per step, and then were further dried by CO_2_. All prepared dried samples were sputter-coated with platinum and analyzed using SEM at an accelerating voltage of 5.0 kV under the high vacuum.

### 2.8. Statistical Analysis

All measurements were conducted in triplicate independent experiments. The parameters of kinetic inactivation models were calculated by the USDA integrated pathogen modeling program (IPMP) [46]. The effect of the different physical structures of food materials was evaluated by one–way analysis of variance and Duncan’s multiple comparison tests at a 5% significant level or independent *t*-tests using the statistical software (17.0 version, SPSS Inc., Chicago, IL, USA). Parameters were re-estimated in the statistical SPSS software.

## 3. Results and Discussion

### 3.1. Selection of the Most Heat-Resistant Strain in Wheat Kernels and Flour

**Figure 1:**
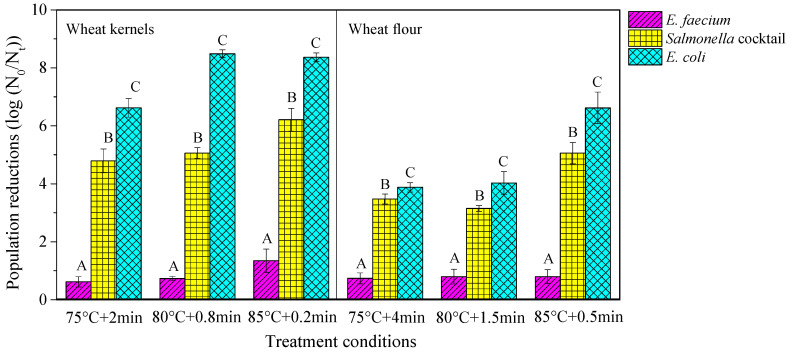
Population reductions (log CFU/g) of *E. faecium* NRRL-2356, *Salmonella* cocktail (*S*. *typhimurium* ATCC 50115 and *S*. *braederup* H9812), and *E. coli* ATCC 25922 in wheat kernels (a_w_ = 0.66) and flour (a_w_ = 0.65) in different temperature–time combination treatment conditions under a fixed heating rate of 5 °C/min in the heating block system (HBS). The mean values over three replicates with the same uppercase letters are not significantly different at *p* > 0.05 by Duncan’s multiple comparison tests among population reductions of different bacteria strains at a fixed treatment condition.

Figure 1 shows the population reductions of *E. faecium*, *Salmonella* cocktail, and *E. coli* when inoculated in wheat kernels (a_w_ = 0.66) and flour (a_w_ = 0.65) at three treatment conditions with a fixed heating rate of 5 °C/min in the HBS. The results showed that heat resistances of all strains were significantly different (*p* < 0.05) at all temperature–time combinations when inoculated in wheat kernels and flour. The population reductions of *E. faecium* were significantly lower (*p* < 0.05) than those of *Salmonella* cocktail and *E. coli* whether the bacteria were inoculated in wheat kernels or flour. In addition, *E. coli* showed a significantly lower heat resistance (*p* < 0.05) than *Salmonella* cocktail in wheat kernels and flour, which was contrary to the result in almond [32] and red pepper powder [20]. These different results may be caused by different main components in inoculated foods. Therefore, *E. faecium,* with the highest heat resistance, was chosen to investigate the influences of food matrices with the same components and different physical structures on microbial inactivation kinetics in further experiments.

### 3.2. Influence of Graininess and Powder Structures of Food Matrices on Heat Resistance of E. faecium

**Figure 2:**
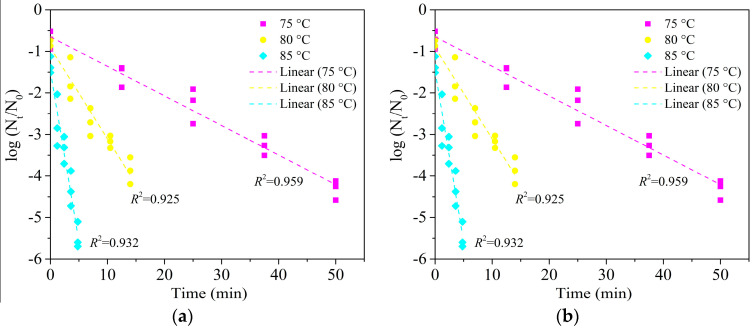
Inactivation kinetic curves of *E. faecium* in wheat granules (a_w_ = 0.66) (**a**) and flour (a_w_ = 0.65) (**b**) at 75, 80 and 85 °C under a fixed heating rate of 5 °C/min in the HBS.

The population level in the inoculated wheat kernels (a_w_ = 0.66) and flour (a_w_ = 0.65) were 8.84 ± 0.20 and 9.23 ± 0.34 log CFU/g, respectively (Table 1). Inactivation kinetics of *E. faecium* inoculated in wheat granules (*a_w_* of 0.66) and flour (a_w_ of 0.65) are shown in Figure 2. When heated to a fixed temperature, there were different inactivation degrees of microorganisms (0.24–1.39 log CFU/g), and the reduced population of *E. faecium* may not affect the thermal inactivation in wheat kernels and flour [11]. Survival data of *E. faecium* inoculated in wheat kernels and flour fitted well to both primary models with low average root mean square error (RMSE) (<0.5 log CFU/g) and showed a better fit to the linear model for both food matrices with smaller *AIC* (Table 2).

The *D*-values of 18.59 ± 1.49, 6.49 ± 0.56, and 2.08 ± 0.21 min for *E. faecium* inoculated in wheat flour were slightly lower than those of 25.5 ± 1.2, 11.4 ± 1.0, and 2.7 ± 0.2 min in wheat flour (a_w_ = 0.6) at 75, 80 and 85 °C, respectively [18]. The differences in *D*-values at the same heating temperatures may be due to different a_w_, heating rate, isothermal treatments, and protocols of bacterial inoculation [15,20,33].

As shown in Table 2, the *D*-values of *E. faecium* in wheat flour were higher than those in kernels at temperatures of 75–85 °C. The results showed a higher heat resistance for *E. faecium* in wheat flour inoculum (powder structure), which was similar to the result of heat resistance of *S. enteritidis* PT 30 when inoculated in wheat kernels and flour [12]. That is, *S. enteritidis* PT 30 had a higher *D*-value of 15.1 ± 0.7 min when inoculated in wheat flour compared to that of 8.9 ± 0.4 min when inoculated in wheat kernels at the same a_w_ of 0.40. The reason for the differences in heat resistance of microorganisms inoculated in the materials with the same composition and a_w_ may be caused by the different physical structures of the inoculated materials [20]. For example, when inoculated in food matrices, the microorganisms were surrounded by flour with smaller particles but loose structure while only inoculated on the surfaces of wheat kernels. The difference in the two physical structures of food matrices contributes to the various heat transfer rate between the food matric and microorganism during the heating process. Therefore, microorganisms on the surface of wheat kernels (graininess structure) were easily inactivated due to the direct heat transfer to microorganisms [20].

### 3.3. Influence of Graininess and Paste Structures of Food Matrices on Heat Resistance of E. faecium

**Figure 3:**
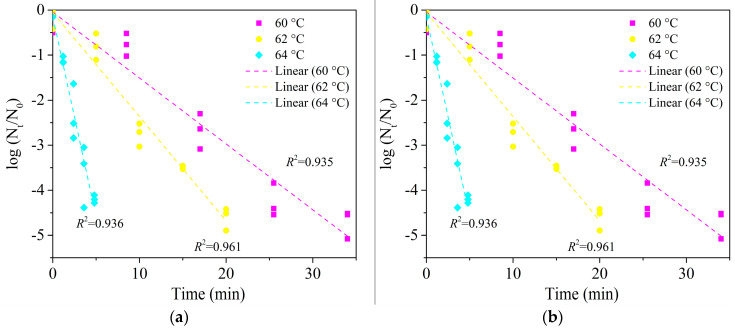
Inactivation kinetic curves of *E. faecium* in wheat granules (**a**) (a_w_ = 0.99) and dough (**b**) (a_w_ = 0.99) at 60, 62 and 64 °C under a fixed heating rate of 5 °C/min in the HBS.

The population levels of the inoculated wheat kernels and dough at a_w_ of 0.99 were 9.37 ± 0.17 and 9.34 ± 0.06 log CFU/g, respectively (Table 1). Inactivation kinetics of *E. faecium* inoculated in wheat granules (a_w_ of 0.99) and dough (a_w_ of 0.99) are shown in Figure 3. There were different degrees of microorganism reduction (0.12–0.51 log CFU/g) at three fixed temperatures, and the reduced population of *E. faecium* may not affect the thermal inactivation in wheat kernels and dough [11]. Survival data of *E. faecium* inoculated in wheat kernels and dough fitted well to both primary models with low average RMSE (<0.5 log CFU/g) and showed a better fit to the linear model for wheat kernels with smaller *AIC* than the Weibull model for wheat dough (Table 3).

As listed in Table 3, the *D*-values of *E. faecium* in wheat dough were higher than those in kernels at temperatures of 60–64 °C. The results showed a higher heat resistance for *E. faecium* in wheat dough (paste structure). The reason for the difference of heat resistances of *E. faecium* inoculated in materials at the same components and a_w_ may be caused by the porous and irregular physical structures of wheat dough due to its gelatinization [21] compared to the compact structure of wheat kernel during HBS treatments. This structural change may be contributing the microorganisms easier surrounded by starch-protein matrix, resulting in a worse heat transfer with a better adaption of *E. faecium* to heat stress.

### 3.4. Microstructure Observation by SEM

**Figure 4:**
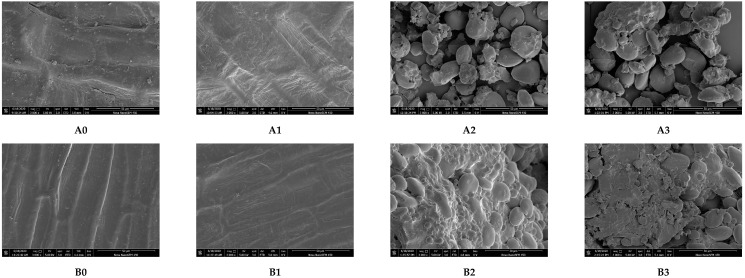
Scanning electron microscope (SEM) images of wheat samples with three physical structures (All images were 3000× magnification). (**A0**,**A2**,**B0**,**B2**) were control samples without heating for wheat kernel surface (a_w_ = 0.66), wheat flour, wheat kernel surface (a_w_ = 0.99), and wheat dough, respectively. (**A1**,**A3**) were treatment samples for wheat kernel surface (a_w_ = 0.66) and wheat flour heating at 80 °C for 5 min in HBS. (**B1**,**B3**) were treatment samples for wheat kernel surface (a_w_ = 0.99) and wheat dough heated at 62 °C for 5 min in HBS.

The control samples of wheat kernels (Figure 4A0,B0) had uneven surfaces. After heat treatment, some crumples were observed on the surfaces of wheat kernel samples (Figure 4A1,B1). The elliptical A- and B-type starch granules with various sizes were observed in wheat flour (Figure 4A2,A3), and starch granules had no obvious changes before and after treatment due to the short time treatment of within 5 min. A starch–protein matrix with a porous structure was observed in control samples of wheat dough (Figure 4B2), and some starch granules began to swell after heat treatment (Figure 4B3).

**Figure 5:**
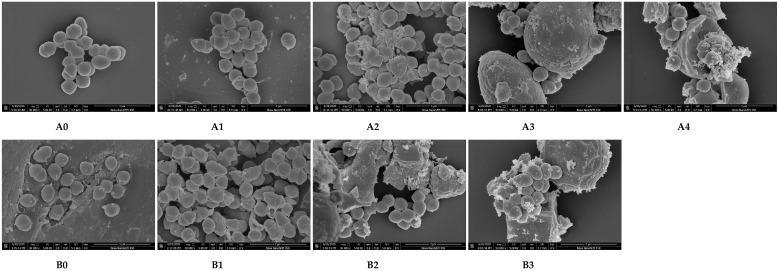
SEM images of *E. faecium* inoculated in wheat samples with three physical structures (all images were 50,000× magnification). (**A0**) was the liquid suspension of *E. faecium* without any treatment. (**A1**,**A3**,**B0**,**B2**) were control cell samples without heating when inoculated in wheat kernel surface (a_w_ = 0.66), wheat flour, wheat kernel surface (a_w_ = 0.99), and wheat dough, respectively. (**A2**,**A4**) were treatment samples inoculated in wheat kernel surface (a_w_ = 0.66) and wheat flour heated at 80 °C for 5 min in HBS. (**B1**,**B3**) were treatment samples inoculated in wheat kernel surface (a_w_ = 0.99) and wheat dough heated at 62 °C for 5 min in HBS.

The liquid suspension of *E. faecium* (Figure 5A0) and its inoculation in different wheat samples without treatment (Figure 5A1,A3,B0,B2) had complete and clear profiles. After heat treatment, the *E. faecium* had different degrees of shrinkages (Figure 5A4,B3) and damages (Figure 5A2,B1). Some cells started to shrink in wheat flour (Figure 5A4), while most cells were slightly shrunk in the wheat dough (Figure 5B3). While inoculated in wheat kernels, most of the cells were severely shrunk and damaged (Figure 5A2,B1). Therefore, the changes in cells mentioned above indicated that the *E. faecium* inactivation was influenced by the physical structure of the food matrices and was more easily inactivated in the graininess structure under the same given heating condition.

### 3.5. Influence of Physical Structures of Food Matrices on Heat Resistance of E. faecium

When food matrices had the same basic compositions, and a_w_, their particle sizes affected the heat resistance of the *E. faecium*, which may be caused by their different densities and heat transfer rates [12,20]. In the Weibull model, almost all shape parameters of trials were closed to 1, excluding wheat flour at 85 °C and wheat dough, implying that the inactivation rate of *E. faecium* was not influenced by temperature changes at the given conditions. A *p*-value > 1 for wheat flour at 85 °C indicated a shoulder effect, a direct lethal effect of the thermal treatment than the accumulation effect, on *E. faecium*. *P*-values < 1 for wheat dough showed a tailing behavior for inactivation of *E. faecium* in the wheat dough, revealing that the sensitive bacterium inactivated first and the remaining could be the strong ones or adapting to the heat stress. These shape values of models shown in Table 2 and Table 3 were influenced by the physical structure of food matrices. Particularly, the changes of physical structures caused by gelatinization of wheat dough (Figure 4B3) during heating provided more protection for *E. faecium* due to irregular shape changes. These changes strengthened the heat resistance of *E. faecium* by adapting to the thermal pressure gradually. Thus, a nonlinear inactivation curve was better fitting with an upward concavity trend (*p* < 1) for *E. faecium* in this condition compared to wheat kernels and flour. The physical structure changes caused by gelatinization may affect the heat transfer ability from food matrices to *E. faecium* and a heat resistance of *E. faecium*. A comprehensive understanding of the influence of changes of physical structures on microbial thermal resistance requires further investigations [12,21].

**Figure 6:**
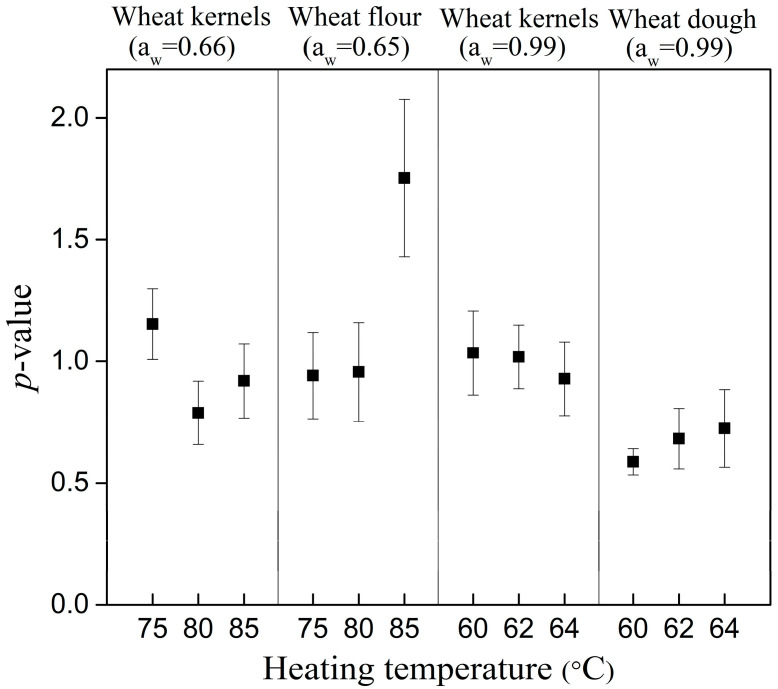
The shape parameter *p* and associated 95% confidence interval as a function of heating temperature for *E. faecium* in food matrices with different physical structures.

To investigate the influence of physical structures of food matrices on the heat resistance of *E. faecium*, the *δ* value was re-estimated by the Weibull model with a single *p*-value. This approach can eliminate the structural correlation between the two parameters of the Weibull models [26]. As shown in Figure 6, the a_w_, temperature and physical structures of food matrices had no great influence on the *p*-value with an *R*^2^ of 0.264. This observation is in agreement with the result of Couvert et al. for *Bacillus pumilus* [44] and Fernandez et al. for *Bacillus cereus* spores [47], but in disagreement with Fernandez et al. for *Listeria monocytogenes* [48] and Zhang for *E*. *coli* ATCC 25922 [20]. According to references [20,44,49], Weibull models with a fixed shape parameter can be studied to compare the heat resistance of bacterial by re-estimated *δ*’-value despite the effect of environmental factors. In this study, re-estimated *δ*’-values by a constant *p*-value (*p* = 0.96) from whole Weibull curves were compared to study the single effect of physical structures of food matrices on heat resistances of *E. faecium* (Table 1). The result showed that the average inoculation concentrations of *E. faecium* had no significant difference (*p* > 0.05) when the physical structures of food matrices varied, but these structural changes had a significant impact on the heat resistance of *E. faecium* (*p* < 0.05). These results also suggest that cells attach concentrations in food matrices were similar at any physical structures, and a less heat resistance of *E. faecium* was observed in wheat kernels compared to wheat flour or dough at any given temperatures.

The above results showed that the changes of physical structures in food matrices had an impact on the heat resistance of the microorganism. With changing physical structures of food matrices, the microorganisms made different response changes under the heat stress. It seems that a more complex survival environment (starch or starch-protein matrix, as shown in Figure 4A3,B2) resulted in a better adaption of microorganisms to heat stress. These adaption differences may be caused by a worse heat transfer between food matrices and microorganisms [20]. Some other studies have pointed out that different compositions of food matrices affect the heat resistance of inoculated microorganisms due to their a_w_ changing at high temperatures [13,14,19]. For example, *S. enteritidis* PT 30 had a lower *D*_80 °C_-values of 6.9 min in wheat flour corresponding to an increase a_w_ from 0.45 to 0.80 while higher *D*_80 °C_-values of 17.0 min in peanut butter corresponding to a decrease a_w_ from 0.45 to 0.04, respectively. Thus, the different heat resistances of *E. faecium* inoculated in materials with different physical structures may be attributed by various compositions in different attachment locations with outer grain layers (when inoculated on kernel surfaces) and mixtures with whole wheat kernels (when inoculated in wheat flour or dough) since wheat kernels are made of heterogeneous compositions [5,50]. However, the above speculation needs to be verified by detecting the changes of a_w_ with temperature changing in different attachment locations.

An effective pasteurization method could be considered in terms of the specific physical structure of the material. The result obtained in this study indicated that a better thermal inactivation effect could be achieved in wheat kernel structure comparing with wheat flour and dough. Moisture tempering, a process of separating the bran and flour easily by adjusting the MC of wheat [5], is one of the processes during wheat to wheat-based food processing. The relatively high MC (15.0–16.0%) of wheat kernels in the tempering process could be beneficial to the effective inactivation of microorganisms [51,52]. Therefore, a pasteurization process after moistening could be suggested in industrial applications to control the target pathogen in wheat-based food for achieving acceptable food safety and product quality [32,52].

## 4. Conclusions

This study demonstrated that *E. faecium* NRRL-2356 could be used as a surrogate for *Salmonella* cocktail in wheat kernels and its derivatives due to its most heat-resistant characteristics. The heat resistance of *E. faecium* was influenced by the physical structures of food matrices. *E. faecium* showed less heat-resistant behavior in wheat kernel structural conditions. The results also suggested that the moistening stage for wheat kernel structure may be suitable for pasteurization to ensure the hygiene and safety of wheat kernels.

## Figures and Tables

**Table 1:** Basic properties of materials and the effect of the different physical structure of food matrices on the heat resistance of *E. faecium* NRRL-2356.

Food Matrices	Dry Wheat Kernels	Wheat Flour	Wet Wheat Kernels	Wheat Dough
MC (% w.b.)	11.25 ± 0.09 ^a 1^	11.39 ± 0.09 ^a^	38.45 ± 0.04 ^b^	38.54 ± 0.07 ^b^
a_w_ at 25 °C	0.66 ± 0.01 ^a^	0.65 ± 0.01 ^a^	0.99 ± 0.01 ^b^	0.99 ± 0.01 ^b^
Density in TDT cell (g/mL)	1.33 ± 0.03 ^a^	0.71 ± 0.02 ^b^	1.27 ± 0.01 ^c^	0.91 ± 0.03 ^d^
Inoculation concentration (log CFU/g)	8.83 ± 0.20 ^a^	9.23 ± 0.53 ^a^	9.37 ± 0.15 ^a^	9.31 ± 0.16 ^a^
*z*-value (°C)	9.33 ± 0.81 ^a^	10.71 ± 0.60 ^b^	4.94 ± 0.04 ^c^	5.64 ± 0.25 ^d^
*δ*’_75 °C_ (min) ^2^	13.91 ± 1.08 ^A^	17.67 ± 0.85 ^B^		
*δ*’_80 °C_ (min)	4.13 ± 0.12 ^A^	6.30 ± 0.39 ^B^		
*δ*’_85 °C_ (min)	1.12 ± 0.12 ^A^	2.41 ± 0.18 ^B^		
*δ*’_60 °C_ (min)			6.86 ± 0.54 ^A^	14.90 ± 0.70 ^B^
*δ*’_62 °C_ (min)			4.28 ± 0.49 ^A^	6.20 ± 0.32 ^B^
*δ*’_64 °C_ (min)			1.07 ± 0.11 ^A^	2.85 ± 0.11 ^B^

^1^ All measures were expressed as means ± standard deviation(SD) in triplicate independent experimental data. Means with the same lowercase letter are not significantly different at *p* > 0.05 by Duncan’s multiple comparison tests. Means with the same uppercase letter are not significantly different at *p* > 0.05 by independent samples *t*-tests. ^2^ Re-estimated *δ*’-values of Weibull model definite with single *p*-value (0.96) re-evaluated from the whole set of kinetics for *E. faecium* NRRL-2356. TDT: thermal death time.

**Table 2:** Parameter estimates and RMSE for the primary thermal inactivation models of *E. faecium* NRRL-2356 inoculated in wheat kernels (a_w_ = 0.66) (a) and flour (a_w_ = 0.65) and heated at three temperatures with a fixed heating rate of 5 °C/min in the heating block system (HBS) *.

Material	Temperature (°C)	Linear Model			Weibull Model			
		*D*-Value (min)	RMSE (log CFU/g)	*AIC*	*δ* (min)	*p*	RMSE (log CFU/g)	*AIC*
Wheat kernels	75	14.04 ± 0.78	0.27	−33.461	16.72 ± 2.56	1.15 ± 0.15	0.27	−30.894
	80	4.56 ± 0.35	0.32	−28.485	3.31 ± 0.86	0.79 ± 0.13	0.31	−26.998
	85	1.22 ± 0.09	0.39	−22.437	1.06 ± 0.29	0.91 ± 0.15	0.40	−18.784
Wheat flour	75	18.59 ± 1.49	0.38	−23.014	17.13 ± 4.71	0.94 ± 0.18	0.40	−19.147
	80	6.49 ± 0.56	0.44	−19.133	6.45 ± 1.89	0.99 ± 0.20	0.45	−15.134
	85	2.08 ± 0.21	0.40	−21.498	3.28 ± 0.41	1.75 ± 0.32	0.31	−26.794

* All parameter estimates are means ± SD over three independent experimental replications, and root mean square error (RMSE) were estimated separately for each data set and determined by integrated pathogen modeling program (IPMP) software. *AIC:* Akaike Information Criterion.

**Table 3:** Parameter estimates and RMSE for the primary thermal inactivation models of *E. faecium* inoculated in wheat kernels and dough at a_w_ of 0.99 and heated at three temperatures with a fixed heating rate of 5 °C/min in the HBS *.

Material	Temperature (°C)	Linear Model			Weibull Model			
		*D*-Value (min)	RMSE (log CFU/g)	*AIC*	*δ* (min)	*p*	RMSE (log CFU/g)	*AIC*
Wheat kernels	60	6.84 ± 0.48	0.48	−16.172	7.22 ± 2.00	1.03 ± 0.17	0.50	−12.235
	62	4.33 ± 0.23	0.34	−26.343	4.45 ± 0.92	1.02 ± 0.13	0.36	−22.371
	64	1.13 ± 0.08	0.41	−21.149	1.00 ± 0.28	0.93 ± 0.15	0.42	−17.431
Wheat dough	60	18.04 ± 1.39	0.40	−21.758	6.59 ± 1.48	0.59 ± 0.04	0.20	−39.236
	62	7.16 ± 0.66	0.52	−13.509	3.52 ± 1.44	0.68 ± 0.12	0.46	−14.930
	64	3.23 ± 0.33	0.57	−11.019	1.82 ± 0.84	0.72 ± 0.16	0.54	−9.944

* All parameter estimates are means ± SD over three independent experimental replications, and RMSE were estimated separately for each data set and determined by IPMP software.
