# Correction: Lin et al. Effect of Physical Structures of Food Matrices on Heat Resistance of *Enterococcus faecium* NRRL-2356 in Wheat Kernels, Flour and Dough. *Foods* 2020, *9*, 1890

**DOI:** 10.3390/foods10081751

**Published:** 2021-07-29

**Authors:** Biying Lin, Yufei Zhu, Lihui Zhang, Ruzhen Xu, Xiangyu Guan, Xiaoxi Kou, Shaojin Wang

**Affiliations:** 1 College of Mechanical and Electronic Engineering, Northwest A&F University, Yangling 712100, China; linbiying@nwafu.edu.cn (B.L.); 1000005296@ujs.edu.cn (L.Z.); ruzhen_xu@nwafu.edu.cn (R.X.); xiangyuguan@nwafu.edu.cn (X.G.); kouxiaoxi@nwsuaf.edu.cn (X.K.); 2 College of Animal Science and Technology, Northwest A&F University, Yangling 712100, China; zhuyufei@nwsuaf.edu.cn; 3 Department of Biological Systems Engineering, Washington State University, Pullman, WA 99164-6120, USA

The authors make the following corrections to their published paper [1]. In the Results and Discussion section, Figure 2b and Figure 3b were misplaced, making them identical to Figure 2a and Figure 3a, respectively. The corresponding figures are corrected in this correction.

The corrected Figure 2 should be as follows:

**Figure 2:**
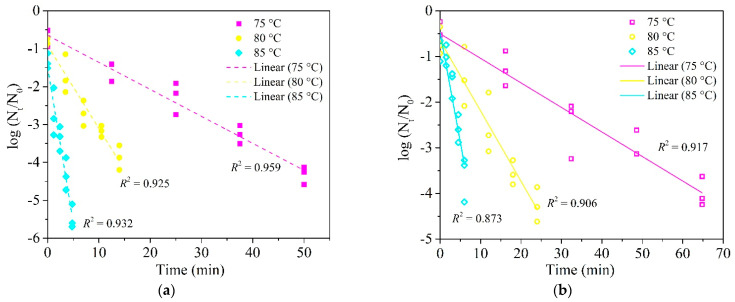
Inactivation kinetic curves of *E. faecium* in wheat granules (a_w_ = 0.66) (**a**) and flour (a_w_ = 0.65) (**b**) at 75, 80 and 85 °C under a fixed heating rate of 5 °C/min in the HBS.

The corrected Figure 3 should be as follows:

**Figure 3:**
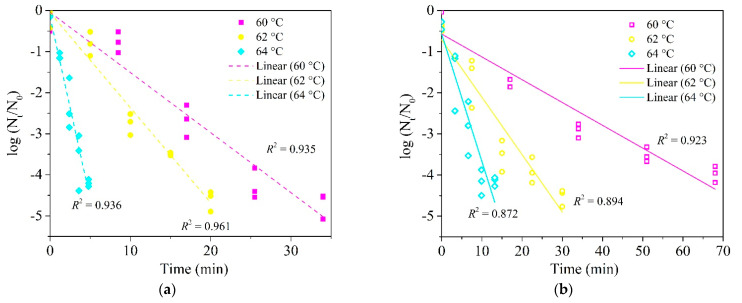
Inactivation kinetic curves of *E. faecium* in wheat granules (**a**) (a_w_ = 0.99) and dough (**b**) (a_w_ = 0.99) at 60, 62 and 64 °C under a fixed heating rate of 5 °C/min in the HBS.

The authors apologize for any inconvenience caused to the readers by these changes. The changes do not affect the scholarly results. The original manuscript will be updated online.
